# Predictors of Caregiver Burden in Caregivers of Bedridden Patients

**DOI:** 10.1097/jnr.0000000000000297

**Published:** 2019-05-20

**Authors:** Aysun BEKDEMİR, Nesrin İLHAN

**Affiliations:** 1 MSc, Specialist Nurse, Home Health Services Coordination Center, Üsküdar State Hospital, Istanbul, Turkey; 2 PhD, Assistant Professor, Faculty of Health Sciences, Department of Nursing, Bezmialem Vakif University, Istanbul, Turkey.

**Keywords:** caregiving, caregiver burden, caregiver health, home care, nurse

## Abstract

**Background:**

Caregivers are at risk of experiencing caregiver burden. It is therefore important to determine the caregiver burden of caregivers who provide care to bedridden patients and related factors.

**Purpose:**

The aim of this study was to determine the caregiver burden of caregivers who provide care to bedridden patients and the factors that impact this burden.

**Methods:**

This cross-sectional study was executed at a state hospital in Istanbul, Turkey, on bedridden patients registered in the home healthcare unit and their caregivers. During study period, the researchers made 312 visits to patients and their caregivers. A sociodemographic questionnaire, the Burden Interview, and the Katz Index of Independence in Activities of Daily Living were used to collect data. Descriptive statistics, an independent sample *t* test, one-way analysis of variance, and stepwise multiple regression analysis were used for data analysis.

**Results:**

The participants reported a moderate level of caregiver burden. Existing caregiver health problems, caregiver employment status, the ability of the caregiver to maintain his or her own good health, type of home, and the degree of patient dependence in terms of activities of daily living were each found to be significant predictors of caregiver burden.

**Conclusions/Implications of Practice:**

The support provided to caregivers by home healthcare units is important in terms of protecting the physical, mental, and social health conditions of caregivers and preventing the exacerbation of caregiver burden.

## Introduction

Bedridden patients are patients who stay in bed for short or long periods for various reasons, including chronic illnesses, old age, and disability. Bedridden patients cannot perform self-care and medical care partially or completely and need the help of others. Bedridden patients are usually cared for by family members, paid caregivers, and/or health professionals (Handicap International, n.d.; Vieira et al., 2015).

Family caregivers are defined as relatives and friends who provide care free of charge to individuals with chronic or debilitating conditions (Collins & Swartz, 2011; Sanuade & Boatemaa, 2015). Family members play important roles in the care of the sick and those unable to take care of their own needs (Adelman, Tmanova, Delgado, Dion, & Lachs, 2014; Chiou, Chang, Chen, & Wang, 2009). Providing care adversely affects the health and quality of life of the caregiver (Bauer & Sousa-Poza, 2015; Jeong, Myong, & Koo, 2015; Rha, Park, Song, Lee, & Lee, 2015). Caregivers are likely to spend less time with their family and friends, experience increased levels of emotional stress, and neglect self-care activities such as getting a good night's sleep, exercising, and healthy eating (Collins & Swartz, 2011).

Caregivers are at risk of caregiver burden (Chang, Chiou, & Chen, 2010; Chiou et al., 2009; Roopchand-Martin & Creary-Yan, 2014). Caregiver burden is defined as a multidimensional response to perceived stress and negative assessments that derive from providing care to a sick person (Kim, Chang, Rose, & Kim, 2012). The risk factors that have been identified in the literature as affecting caregiving burden include being female, having a lower level of education, living in the same house with the care recipient, providing care for long hours, having depression, being socially isolated, being under financial stress, and having no choice but to be a caregiver (Adelman et al., 2014). Caregiver burden threatens the physical, psychological, emotional, and functional health of caregivers (Bauer & Sousa-Poza, 2015; Etters, Goodall, & Harrison, 2008; Ma, Lu, Xiong, Yao, & Yang, 2014). Moreover, caregiver burden is known to be a significant predictor of quality of life (Jeong et al., 2015; Rha et al., 2015).

Researchers have found that caregiver burden is affected by many factors related to providing care. The health of both caregivers and care recipients impact strongly on caregiver burden (Rha et al., 2015; Sanuade & Boatemaa, 2015). The health of persons receiving care and their degree of dependence affect caregiver burden (Abdollahpour, Noroozian, Nedjat, & Majdzadeh, 2012; Conde-Sala, Garre-Olmo, Turró-Garriga, Vilalta-Franch, & López-Pousa, 2010; Zaybak, Güneş, Günay İsmagloğlu, & Ülker, 2012). Furthermore, caregiver burden affects the level of well-being of the caregiver and, as a consequence, reduces the caregiver's ability to provide good care (Collins & Swartz, 2011; Sanuade & Boatemaa, 2015). Chang et al. (2010) found that the mental health and caregiver burden of care providers were related to the health problems experienced by caregivers. Women make up the large majority of caregivers in family settings (Jeong et al., 2015; Rha et al., 2015; Yikilkan, Aypak, & Görpelioğlu, 2014). Whereas some research has identified gender as a significant predictor of caregiver burden (Brodaty et al., 2014; Etters et al., 2008; Sousa et al., 2016), some have found no correlation between gender and caregiver burden (Orak & Sezgin, 2015; Roopchand-Martin & Creary-Yan, 2014; Sanuade & Boatemaa, 2015). Chiou et al. (2009) reported that caregivers with poor social support and family functions have a higher level of caregiver burden and that perceived social support is a better indicator of caregiver burden than the social support actually received. Furthermore, studies have shown that living in the same house with a patient (Adelman et al., 2014; Conde-Sala et al., 2010) and having a direct role in that patient's physical care (Sanuade & Boatemaa, 2015) increase caregiver burden.

Professional healthcare providers have an important impact on the health and well-being of caregivers (Yikilkan et al., 2014). Nurses may engage in training primary caregivers and support caregivers by aiding in care-related activities. Thus, nurses have an important role to play in lessening the caregiver burden of care providers (Schulz & Sherwood, 2008). Nurses are best positioned to make early diagnoses of caregiver burden and to help caregivers avoid/minimize the adverse effects of caregiving (Etters et al., 2008). Nurses should observe caregivers during their home visits and evaluate them in terms of caregiver burden risk (Etters et al., 2008; Yikilkan et al., 2014). When caregiver burden is identified, the perceptions of caregivers regarding the burden that they are taking on may be eased with appropriate interventions (Etters et al., 2008). Thus, for all of the above-stated reasons, it is important to identify caregiver burden and its related factors, to ensure that caregivers receive support, and to develop, organize, and implement caregiver-burden prevention programs. Therefore, the purpose of this study was to determine the caregiver burden of individuals who provide care to bedridden patients and the factors that impact this burden. This study is significant in that it was conducted in Turkey with a large sample and because many factors affecting caregiver burden were examined as variables. The study sought answers to the following research questions:

- What is the relationship between the sociodemographic characteristics of caregivers and their caregiver burden?
- What is the relationship between the way caregivers provide care and their caregiver burden?
- What is the relationship between the degree of dependency of bedridden patients and the caregiver burden of their caregivers?
- Are caregiving-related factors predictive of caregiver burden?

## Methods

### Design and Data Collection

This study used a cross-sectional design and was executed at a state hospital in Istanbul, Turkey, between January and April 2014. The participants were bedridden patients who were registered in the hospital's home healthcare unit and their caregivers.

### Participants

Home healthcare services in Turkey are given by the Ministry of Health, municipalities and private organizations. Home healthcare units of Ministry of Health work in affiliation with the hospitals. Individuals who require services from these units at home are provided examination, testing, treatment, medical care, rehabilitation, and social and psychological support services during home visits. The costs of these services are covered by the social security administration. This study targeted the caregivers of bedridden patients registered in the home healthcare unit of a state hospital in Anatolian Istanbul (*N* = 4,500). The formula that was used to determine the necessary sample size on the basis of a finite population determined that a minimum sample size of 251 bedridden patients and caregivers was required (Sümbüloğlu & Sümbüloğlu, 2002).


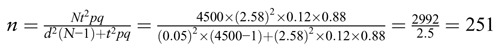


Abbreviations used in the formula:

*N* = Number of individuals in the target population
*n* = Number of individuals to be sampled
*p* = Frequency of occurrence of the event to be investigated (probability)
*q* = Frequency of nonoccurrence of the events to be investigated (1 *p*)
*t* = Theoretical value in the *t* table at a certain degree of freedom and at the determined error level
*d* = The desired deviation according to the occurrence frequency of an event
*N* = 4,500 (number of patients registered at home health units in 2013)
*p* = .12 (the proportion of disabled in Turkey; Turkish Statistical Institute, 2002)
*q* = 0.88
*d* = 0.05
*t* = 2.58 (when *t* = 0.01 in the case of *α* = .01)

The study was conducted on 312 bedridden patients and their caregivers who met the criteria for inclusion. The criteria for inclusion included being responsible for the care of a bedridden patient, providing care for at least 1 month, willing to participate in this research, aged 18 years or older, able to read and write, and able to understand and answer questions. The questionnaires were filled out during face-to-face interviews that were conducted with the participants during a home visit by the researcher. Patient information was obtained from patients and caregivers. Questionnaires with missing data were excluded from the study.

### Ethical Considerations

Verbal and written permission was obtained from the institution in advance. The university's ethics committee granted its approval for this study (September 9, 2013, No. 47). The purpose of this study was explained to the patients and their caregivers, and their written and verbal consent was obtained.

### Measures

#### Demographic variables

The descriptive characteristics of the caregivers (gender, age, educational status, civil status, type of residence, employment status, and income status), their personal health situation, their ability to attend to their own health, their relationship to the patient, the duration that they had been caring for their patient, the areas of caregiving, and the patient's gender, age, and educational status were collected using a standard questionnaire.

#### Caregiver burden

The caregiver burden of the caregivers was measured using the Zarit Burden Interview (BI). In 1980, Zarit, Reever, and Bach-Peterson developed BI as a scale to evaluate the level of stress experienced in providing care to the sick and older adults. The scale questionnaire, which may be filled out either by the caregiver or a researcher, consists of 22 statements on the effect of caregiving on the respondent caregiver's life. Each of the statements is answered using a Likert-type scale, with scores ranging from 0 to 4 (*never*, *rarely*, *sometimes*, *frequently*, and *always*). Studies have indicated an internal consistency coefficient of .87–.94 for the scale and a test–retest reliability of .71. The total possible scores for the BI range from 0 to 88, with 0–20 indicating “no burden,” 21–40 indicating “mild burden,” 41–60 indicating “moderate burden,” and 61–88 indicating “severe burden.” The scale items generally address the social and emotional domains, with a higher total score associated with a greater burden experienced (Zarit et al., 1980). İnci and Erdem (2008), who carried out the validity and reliability studies for the Turkish version of this scale, found a Cronbach's alpha value of .95. The Cronbach's alpha value for the BI found in this study was .90.

#### Functional status

The dependency status of participants was assessed using the Katz ADL was used as Katz Index of Independence in Activities of Daily Living. In 1963, Katz, Ford, Moskowitz, Jackson, and Jaffe developed Katz ADL as a tool to assess basic ADL, and Yardimci (1995) completed the Turkish translation of the index. The index contains six headings, including bathing, dressing, toileting, transferring, continence, and feeding. Each heading has three possible responses: “*dependent*,” “*partially dependent*,” and “*independent*,” which are assigned scores of 1, 2, and 3, respectively. ADL index score totals are assessed as follows: 0–6 = dependent, 7–12 = partial dependence, and 13–18 = independent. The ADL Cronbach's alpha value for this scale found in this study was .92.

### Data Analysis

The study data were analyzed using SPSS 16.0 (SPSS, Inc., Chicago, IL, USA). Descriptive statistics (numbers, percentages, means, and standard deviations) were used in the analysis. The one-sample Kolmogorov–Smirnov test was used to assess normal distribution, and the *p* value was found to be > .05. As the data displayed normal distribution, parametric tests were employed in the advanced analysis. The parametric tests used included the independent sample *t* test for two independent variables and the one-way analysis of variance for more than two independent variables. Stepwise multiple regression analysis was used to determine the factors affecting caregiver burden. The Durbin–Watson statistic was used to measure autocorrelation. The Durbin–Watson statistic was found to be 2.086. Tolerance was found to be between .85 and .99, and the variance inflation factor was 1.00–1.17. Tolerance and variance inflation factor values were both found to be within acceptable limits. The results were found to be within the 95% confidence interval, and significance was assessed as *p* **<** .05.

## Results

### Sample Characteristics

Two thirds (65.1%) of the caregiver participants were women, 57.4% were 36–55 years old, and 60.6% had received over 8 years of training. In addition, 72.8% were married, 30.4% were employed, 64.1% earned an income that was roughly equal to their expenditures, and a large majority (80.1%) lived in apartment residences. Slightly over two fifths (42.6%) self-reported as having some kind of health problem, and 57.1% stated that they did not tend to their health needs. It was found that 71.8% were taking care of their parent or sibling, 56.7% had been a caregiver for 2 years or more, and 85.9% said they tended to every need of their patient. In terms of care recipients, 56.7% were women, the large majority (82.4%) were older than 65 years, and 79.5% had an education of 8 years or less (Table 1). The ADL item mean scores of the care recipients varied between 1.16 ± 0.47 and 1.52 ± 0.70, and their ADL total mean score was 7.64 ± 2.94 (Table 2).

**TABLE 1.**
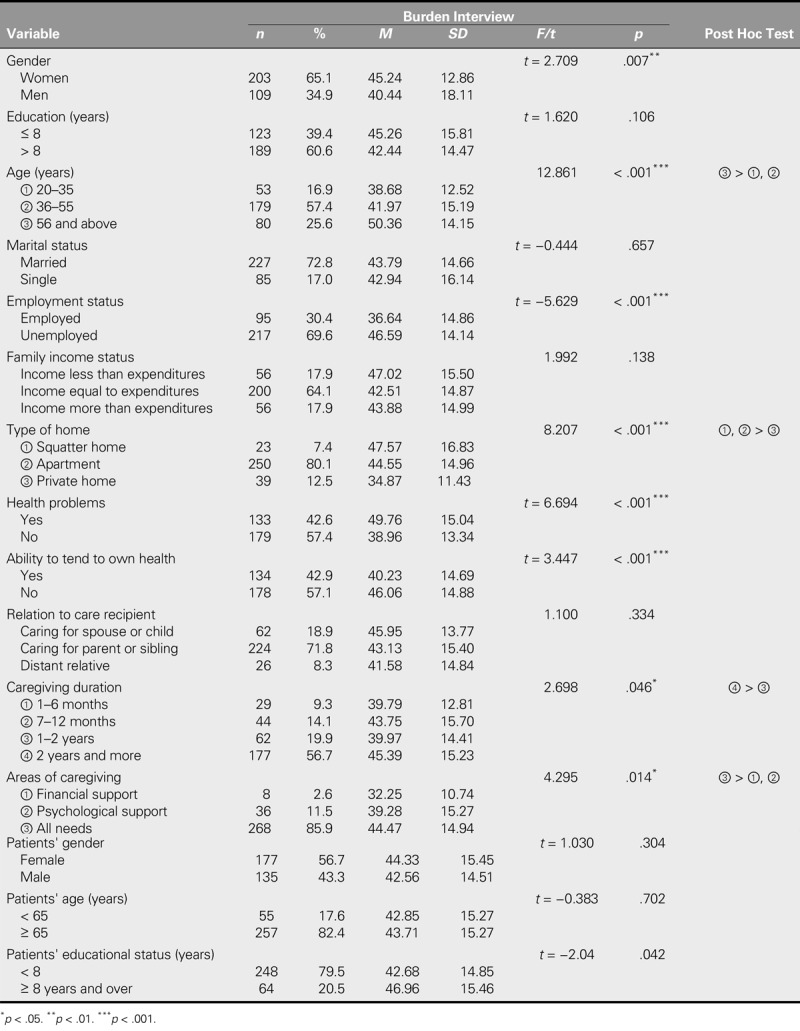
Comparison of the Burden Interview Mean Scores of Caregivers According to Various Personal Characteristics (*N* = 312)

**TABLE 2.**
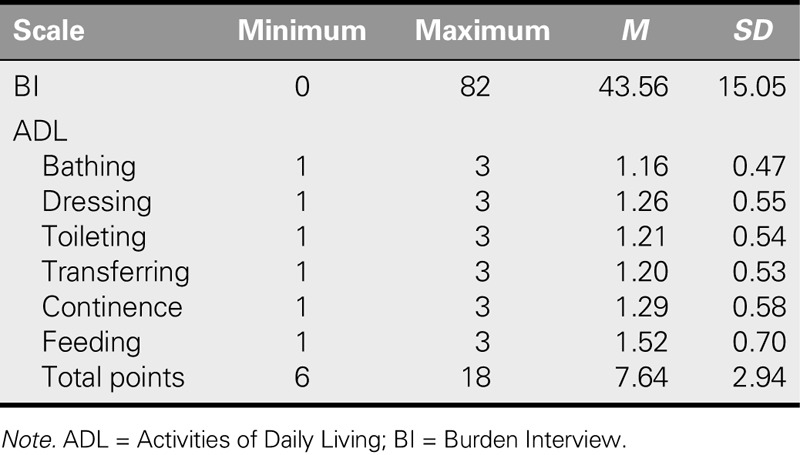
Patients' ADL and BI Mean Scores

### Factors Associated With the Burden of Caregiving

The BI total mean score in this study was 43.56 ± 15.05. The BI mean score of the female caregivers was significantly higher than that of the male caregivers (*p* < .01). The BI mean score of the caregivers who were 56 years old or more was significantly higher than those of caregivers in the other age groups (*p* < .001). The BI mean score of the unemployed caregivers was significantly higher than that of the employed caregivers (*p* < .001). The BI mean score of caregivers living in squatters' homes and apartments was significantly higher than that of caregivers living in private homes (*p* < .001). The BI mean score of caregivers who had health problems was significantly higher than that of caregivers with no health problems (*p* < .001).

Those caregivers who did not tend to their health needs had a BI mean score significantly higher than that of caregivers who did take care of their health needs (*p* < .001). Caregivers who had been tending to their patients for 2 years or more had a BI mean score significantly higher than that of caregivers who had been tending to their patients for 1–2 years (*p* < .05). The BI mean score of caregivers who tended to all of the patients' needs was significantly higher than that of caregivers who provided only financial or psychological support (*p* < .05). The BI mean scores did not display any statistically significant differences based on the level of education, marital status, or family income level of the caregivers or on their relationship to the care recipient (*p* > .05). The BI mean score of the caregivers of patients with a level of education over 8 years was significantly higher than that of caregivers of patients with a level of education of 8 years or less (*p* < .05). No statistically significant differences were found between the BI mean scores of caregivers categorized, respectively, by care recipient gender and age (*p* > .05; Table 1).

In comparing the BI mean scores of patients according to their ADL, the BI mean scores for caregivers of patients who were partially dependent on the caregiver for dressing, continence, and feeding issues were significantly higher than the mean scores for those caregivers who were caring for completely dependent patients (*p* < .01). No statistically significant differences were found between the BI mean scores of caregivers based on the bathing, toileting, and transfer dependency needs of patients (*p* > .05; Table 3).

**TABLE 3.**
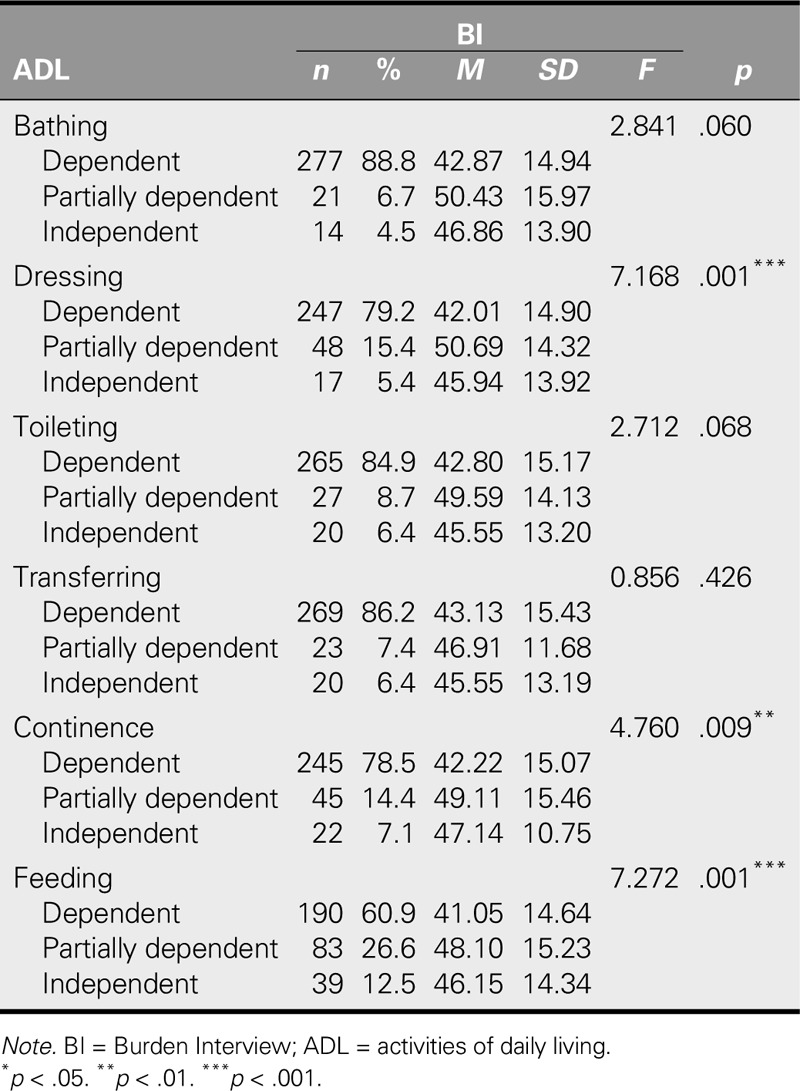
Comparison of Caregivers' BI Mean Scores According to Patients' ADL

### Determinants of the Burden of Caregiving

A stepwise multiple regression analysis was performed to determine the predictors of the burden of caregiving. In the univariate analysis, the variables that had a significant effect on the burden of caregiving were considered as independent variables. Thus, gender, age, employment status, the health problems of the caregiver, the ability of the caregiver to tend to personal health, type of home, duration of care, the areas of care, and the patient ADL score were adopted as the independent variables. It was determined that gender, age, duration of care, and the areas of care of caregivers were not significant predictors of caregiver burden. However, significant relationships were found between burden of caregiving and the caregivers' health problems, employment status, ability to tend to personal health, type of home, and ADL (*R*^2^ = .25, *p* < .001), with 25% of the total variance explained by these variables. The order of significance for the variables ranged from the caregiver's health problems (*β* = .257, *p* < .001) to employment status (*β* = −.225, *p* < .001), type of home (*β* = −.182, *p* < .001), ability to tend to personal health (*β* = −.170. *p* < .001), and the dependency of the patient in ADL (*β* = .147, *p* < .001; Table 4).

**TABLE 4.**
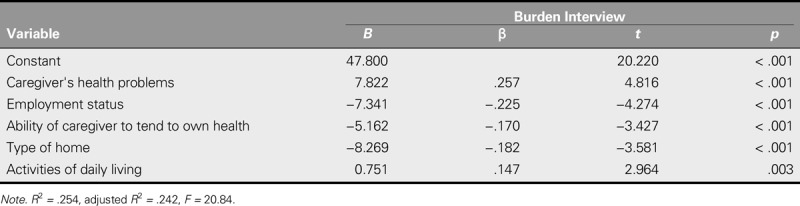
Caregiver Burden Predictors According to Results of Multiple Regression Analysis

The burden of the caregivers in this study was found to be higher in those who had health problems, who were unable to manage their own health, who were not employed, who lived in a squatter home or in an apartment, and who were less dependent on their patients.

## Discussion

The aim of this study was to determine the caregiver burden of individuals who were caring for bedridden patients and the factors that impact this burden. This study was conducted on a sample of bedridden patients who were receiving services from a home healthcare unit and their caregivers. It was determined that the caregivers experienced a moderate level of caregiver burden. Using univariate analysis, a significant relationship was found between caregiver burden and the caregiver's gender, age, employment status, type of home, health problems, ability to tend to personal health, and duration of caregiving and the areas where caregiving was needed as well as the patient's dependency in terms of ADL. Multiple regression analysis showed that caregiver health problems, their employment status, their ability to tend to their own health, their type of home, and the degree of dependence of patients in terms of ADL were each significant predictors of caregiving burden.

The caregiver burden scores for caregivers with health problems were higher than those of caregivers who had no health problems, indicating that caregiver burden increases when caregivers have personal health problems. One study pointed to correlations between health problems and the mental health of caregivers and caregiver burden (Chang et al., 2010). Half of the caregivers in one prior study had at least one chronic health problem (Collins & Swartz, 2011). Caregiving creates a physical and mental burden that adversely affects the health of caregivers (Bauer & Sousa-Poza, 2015; Etters et al., 2008; Kim et al., 2012), and poor personal health disrupts personal quality of life (Jeong et al., 2015). Furthermore, poor caregiver health lowers the quality of care given, increases caregivers' formal demands for healthcare (Bauer & Sousa-Poza, 2015; Collins & Swartz, 2011), and increases healthcare costs (Bauer & Sousa-Poza, 2015). It is important that caregivers are supported by home healthcare units so that they do not feel alone and helpless, do not develop health issues, and do not experience a deterioration in their existing health problems.

The caregiver burden of caregivers who do not tend to their own health is higher than that of caregivers who do. Paying appropriate attention to personal health reduces caregiver burden. The health of caregivers is known to impact caregiver burden strongly (Rha et al., 2015). Chiou et al. (2009) observed that caregivers with a low level of social and functional family support experienced higher levels of caregiver burden. The fact that caregivers allocate time to deal with their own health problems indicates that they receive social support from friends and family when needed. The literature supports that social support reduces the caregiver burden and increases the quality of life of caregivers who provide care for patients with chronic diseases (Atagün, Balaban, Atagün, Elagöz, & Özpolat, 2011).

This study found that the caregiving burden of unemployed caregivers was higher than that of their employed counterparts, indicating that outside employment reduces caregiver burden. A prior study reported that the caregiver burden of employed caregivers is of a lower level compared with that of unemployed caregivers (Sanuade & Boatemaa, 2015). Another study found that self-employed caregivers had lower caregiver burden scores (Roopchand-Martin & Creary-Yan, 2014). As caregivers who hold jobs outside the home cannot serve as primary, full-time caregivers, their caregiving time is shorter than that of caregivers who are not employed, which may explain why employed caregivers have a lower caregiver burden. Chiou et al. (2009) found that caregiver burden increases as the duration of caregiving. In Yeşil, Uslusoy, and Korkmaz (2016), no difference was found in caregiver burden based on employment status.

The caregiver burden of caregivers who live in squatter houses or apartments was shown to be higher than those who live in private houses, indicating that living in a private house reduces caregiver burden. This result indicates that physical circumstances may affect caregiver burden. Istanbul is one of Turkey's most densely populated and most expensive cities. Accordingly, it is likely that people who live in private homes in Istanbul are of a relatively high socioeconomic status. As caregivers at higher economic levels care for their patients in more comfortable physical conditions and are more likely to employ outside help, their caregiver burden may subsequently be less.

Caregivers of patients with higher levels of education were found to have higher burdens of care than those of patients with low levels of education. As patients with higher levels of education have higher life expectancies, they likely have greater expectations from their caregivers. This may increase the burden of care of their caregivers.

This study found that patients were most commonly dependent on their caregivers for bathing and least dependent for feeding, with results showing that the degree of patient dependency was a significant predictor of caregiving burden. As patient dependency lessened, caregiver burden increased. Concurrently, the caregiver burden of individuals caring for patients who were partially dependent because of continence or feeding issues was higher than the burden of those caring for dependent patients. This outcome suggests that the caregivers of partially dependent patients may not have been able to accept the additional dependency-related burdens. As patient dependency increases, caregivers tend to feel that the patients actually do need them and therefore accept the situation, leading to lower levels of perceived caregiver burden. In a study by Taşdelen and Ateş (2012), as patient dependency grew in terms of ADL, the caregiver's emotional burden lessened, which is consistent with the results of this study. However, contrary to the results of this study, other studies have shown that caregiving burden increases as patient dependency rises (Abdollahpour et al., 2012; Kim et al., 2012; Zaybak et al., 2012). It is important that further qualitative studies be conducted to discover the reasons for these outcomes, so that appropriate interventions may be designed to lessen the burden of caregiving.

Women comprised a large majority of caregivers in this study. Other studies have also found that most caregivers are women (Rha et al., 2015; Unver, Basak, Tosun, Aslan, & Akbayrak, 2016; Yeşil et al., 2016). Whereas the univariate analysis revealed a higher level of caregiver burden in female caregivers compared with male caregivers, the multiple regression analysis did not reveal a significant relationship between gender and caregiver burden. Similar to the results of this study, other studies have not detected any significant relationship between gender and caregiver burden (Orak & Sezgin, 2015; Roopchand-Martin & Creary-Yan, 2014; Sanuade & Boatemaa, 2015). However, other studies still have pointed to female caregivers having greater levels of caregiver burden than their male counterparts (Sanuade & Boatemaa, 2015; Sousa et al., 2016; Unver et al., 2016). These results reveal that the burden of caregiving in women may be affected not only by gender but also by normal responsibilities such as housework and childcare, by personal characteristics, by employment status, and by other relevant factors.

Although the burden of caregiving was found to be higher in caregivers aged 56 years and above, age was found not to be a significant determinant of this burden. Similarly, other studies have shown age not to significantly impact the burden of caregiving (Abdollahpour et al., 2012; Roopchand-Martin & Creary-Yan, 2014). Despite this, it is still believed that the older a caregiver is, the higher the caregiving burden may be because of age-related health problems and physical limitations.

It was found that caregivers with 2 or more years of caregiving experience had a higher level of caregiver burden than those with durations of care of 1–6 months and 1–2 years. Similar to the results of this study, Çetinkaya and Karadakovan (2012) found that longer caregiving durations were positively associated with higher caregiver burden. In a study by Yikilkan et al. (2014), caregivers who cared for their patients for more than 3 years had higher levels of depression and anxiety than caregivers with shorter caregiving durations. Regression analysis showed that duration of caregiving was not a significant predictor of the burden of caregiving. This finding is an important outcome, as it shows that caregiver burden is associated with more than only the duration of the caregiving. It may be that long-term caregiving results in higher caregiver burden because of the increases in frequency and severity of physical, mental, and social problems.

In this study, the caregiver burden of caregivers who were required to meet all of the needs of their patients was higher than that of caregivers who met only the financial needs or provided psychological support to their care recipients. Multiple regression analysis found no significant relationship between areas of care and caregiver burden. Mollaoğlu, Özkan Tuncay, and Kars Fertelli (2011) found that those caregivers who met all of the needs of their patients had a higher level of caregiver burden. Moreover, Sanuade and Boatemaa (2015) found that caregivers who provided only financial support and caregivers who received outside financial and physical support had lower levels of caregiver burden. Caregivers who provide patients with only financial or psychological support are not primary caregivers. In these types of cases, the main caregiver is usually another member of the family, and the caregiver's burden is less compared with those who must meet all of their patients' needs.

The cross-sectional approach used in this study limits its generalizability to similar populations only. In addition, the self-report nature of data collection potentially limits the accuracy and generalization of results. The relatively large sample size is a strength of this study. Future studies should consider more complex variables dealing with caregiving as predictive variables (e.g., caregiver-perceived social support, coping strategies, daily care hours, having help available at home, number of caregivers). Furthermore, in line with the results of this study, it is recommended that experimental studies be carried out to evaluate the effectiveness of nursing interventions that are carried out to reduce the caregiver burden of individuals who provide care to bedridden patients.

### Conclusions

The caregivers in this group reported a moderate overall level of caregiver burden. Furthermore, the health status, employment status, ability to tend to personal health matters, and type of home of the caregiver and the degree of dependence of the patient in terms of ADL were all found to be significant predictors of caregiver burden.

## References

[bib1] Abdollahpour I. Noroozian M. Nedjat S. & & Majdzadeh R. (2012). Caregiver burden and its determinants among the family members of patients with dementia in Iran. *International Journal of Preventive Medicine*, 3(8), 544–551. 22973484 PMC3429801

[bib2] Adelman R. D. Tmanova L. L. Delgado D. Dion S. & & Lachs M. S. (2014). Caregiver burden: A clinical review. *JAMA*, 311(10), 1052–1060. 10.1001/jama.2014.304 24618967

[bib3] Atagün M. İ. Balaban Ö. D. Atagün Z. Elagöz M. & & Özpolat A. Y. (2011). Caregiver burden in chronic diseases. *Current Approaches in Psychiatry*, 3(3), 513–552. 10.5455/cap.20110323 (Original work published in Turkish)

[bib4] Bauer J. M. & & Sousa-Poza A. (2015). Impacts of informal caregiving on caregiver employment, health, and family. *Journal of Population Ageing*, 8(3), 113–145. 10.1007/s12062-015-9116-0

[bib5] Brodaty H. Woodward M. Boundy K. Ames D. Balshaw R., & PRIME Study Group. (2014). Prevalence and predictors of burden in caregivers of people with dementia. *The American Journal of Geriatric Psychiatry*, 22(8), 756–765. 10.1016/j.jagp.2013.05.004 24012226

[bib6] Çetinkaya F. & & Karadakovan A. (2012). Investigation of care burden in dementia patient caregivers. *Turkish Journal of Geriatrics*, 15(2), 171–178. (Original work published in Turkish)

[bib7] Chang H. Y. Chiou C. J. & & Chen N. S. (2010). Impact of mental health and caregiver burden on family caregivers' physical health. *Archives of Gerontology and Geriatrics*, 50(3), 267–271. 10.1016/j.archger.2009.04.006 19443058 PMC7114152

[bib8] Chiou C. J. Chang H. Y. Chen I. P. & & Wang H. H. (2009). Social support and caregiving circumstances as predictors of caregiver burden in Taiwan. *Archives of Gerontology and Geriatrics*, 48(3), 419–424. 10.1016/j.archger.2008.04.001 18602706

[bib9] Collins L. G. & & Swartz K. (2011). Caregiver care. *American Family Physician*, 83(11), 1309–1317. 21661713

[bib10] Conde-Sala J. L. Garre-Olmo J. Turró-Garriga O. Vilalta-Franch J. & & López-Pousa S. (2010). Differential features of burden between spouse and adult–child caregivers of patients with Alzheimer's disease: An exploratory comparative design. *International Journal of Nursing Studies*, 47(10), 1262–1273. 10.1016/j.ijnurstu.2010.03.001 20374966

[bib11] Etters L. Goodall D. & & Harrison B. E. (2008). Caregiver burden among dementia patient caregivers: A review of the literature. *Journal of the American Academy of Nurse Practitioners*, 20(8), 423–428. 10.1111/j.1745-7599.2008.00342.x 18786017

[bib12] Handicap International. (n.d.). *Training material—PT protocol for bedridden patients*. Retrieved from https://static.aminer.org/pdf/PDF/000/355/038/therapy_of_bedridden_patients.pdf

[bib13] İnci F. H. & & Erdem M. E. (2008). Validity and reliability of the burden interview and its adaptation to Turkish. *Atatürk University School of Nursing Journal*, 11(4), 85–94. (Original work published in Turkish)

[bib14] Jeong Y. G. Myong J. P. & & Koo J. W. (2015). The modifying role of caregiver burden on predictors of quality of life of caregivers of hospitalized chronic stroke patients. *Disability and Health Journal*, 8(4), 619–625. 10.1016/j.dhjo.2015.05.005 26123859

[bib15] Katz S. Ford A. B. Moskowitz R. W. Jackson B. A. & & Jaffe M. W. (1963). Studies of illness in the aged. The index of ADL: A standardized measure of biological and psychosocial function. *JAMA*, 185(12), 914–919. 10.1001/jama.1963.03060120024016 14044222

[bib16] Kim H. Chang M. Rose K. & & Kim S. (2012). Predictors of caregiver burden in caregivers of individuals with dementia. *Journal of Advanced Nursing*, 68(4), 846–855. 10.1111/j.1365-2648.2011.05787.x 21793872

[bib17] Ma H. P. Lu H. J. Xiong X. Y. Yao J. Y. & & Yang Z. (2014). The investigation of care burden and coping style in caregivers of spinal cord injury patients. *International Journal of Nursing Sciences*, 1(2), 185–190. 10.1016/j.ijnss.2014.05.010

[bib18] Mollaoğlu M. Özkan Tuncay F. & & Kars Fertelli T. (2011). Care burden of care givers of stroke patients and related factors. *Dokuz Eylul University Faculty of Nursing Electronic Journal*, 4(3), 125–130. Retrieved from http://acikerisim.deu.edu.tr/xmlui/bitstream/handle/12345/4595/125-130_mollaoglu.pdf?sequence=1&isAllowed=y (Original work published in Turkish)

[bib19] Orak O. S. & & Sezgin S. (2015). Caregiver burden in family members of cancer patients. *Journal of Psychiatric Nursing*, 6(1), 33–39. 10.5505/phd.2015.02986 (Original work published in Turkish)

[bib20] Rha S. Y. Park Y. Song S. K. Lee C. E. & & Lee J. (2015). Caregiving burden and the quality of life of family caregivers of cancer patients: The relationship and correlates. *European Journal of Oncology Nursing*, 19(4), 376–382. 10.1016/j.ejon.2015.01.004 25795160

[bib21] Roopchand-Martin S. & & Creary-Yan S. (2014). Level of caregiver burden in Jamaican stroke caregivers and relationship between selected sociodemographic variables. *The West Indian Medical Journal*, 63(6), 605–609. 10.7727/wimj.2013.060 25803375 PMC4663964

[bib22] Sanuade O. A. & & Boatemaa S. (2015). Caregiver profiles and determinants of caregiving burden in Ghana. *Public Health*, 129(7), 941–947. 10.1016/j.puhe.2015.05.016 26115592

[bib23] Schulz R. & & Sherwood P. R. (2008). Physical and mental health effects of family caregiving. *American Journal of Nursing*, 108(9, Suppl.), 23–27. 10.1097/01.NAJ.0000336406.45248.4c PMC2791523 18797217

[bib24] Sousa M. F. B. Santos R. L. Turro-Garriga O. Dias R. Dourado M. C. N. & & Conde-Sala J. L. (2016). Factors associated with caregiver burden: Comparative study between Brazilian and Spanish caregivers of patients with Alzheimer's disease (AD). *International Psychogeriatrics*, 28(8), 1363–1374. 10.1017/S1041610216000508 27019317

[bib25] Sümbüloğlu K. & & Sümbüloğlu V. (2002). *Biostatistics* (10th ed, p. 264). Ankara, Turkey: Hatipoğlu Publication (Original work published in Turkish)

[bib26] Taşdelen P. & & Ateş M. (2012). The needs of home care patients and the burdens of their caregivers. *Journal of Education and Research in Nursing*, 9(3), 22–39. (Original work published in Turkish)

[bib27] Turkish Statistical Institute. (2002). *The proportion of disability*. Retrieved from http://www.tuik.gov.tr/PreTablo.do?alt_id=1017

[bib28] Unver V. Basak T. Tosun N. Aslan O. & & Akbayrak N. (2016). Care burden and self-efficacy levels of family caregivers of elderly people in Turkey. *Holistic Nursing Practice*, 30(3), 166–173. 10.1097/HNP.0000000000000148 27078811

[bib29] Vieira H. F. Bezerra A. L. D. Sobreira M. V. S. Ankilma J. B. S. Feitosa D. N. A. & & Paraíba F. S. M. C. (2015). Nursing care patient bedridden in household: A systematic review. *FIEP Bulletin*, 85, 253–257. 10.16887/85.a2.60

[bib30] Yardimci A. E. (1995). *The correlation of the health problems of the elderly teachers who live in Istanbul with their daily life activities and instrumental activities of daily life* (Unpublished master's thesis), University of Istanbul, Turkey (Original work published in Turkish)

[bib31] Yeşil T. Uslusoy E. Ç. & & Korkmaz M. (2016). Examining of the life quality and care burden of those who are looking after the patients suffering from chronic diseases. *Gümüşhane University Journal of Health Sciences*, 5(4), 54–66. (Original work published in Turkish)

[bib32] Yikilkan H. Aypak C. & & Görpelioğlu S. (2014). Depression, anxiety and quality of life in caregivers of long-term home care patients. *Archives of Psychiatric Nursing*, 28(3), 193–196. 10.1016/j.apnu.2014.01.001 24856272

[bib33] Zarit S. H. Reever K. E. & & Bach-Peterson J. (1980). Relatives of the impaired elderly: Correlates of feelings of burden. *Gerontologist*, 20(6), 649–655. 10.1093/geront/20.6.649 7203086

[bib34] Zaybak A. Güneş Ü. Günay İsmagloğlu E. & & Ülker E. (2012). The determination of burden care of caregivers for bedridden patients. *Journal of Anatolia Nursing and Health Sciences*, 15(1), 48–54. (Original work published in Turkish)

